# Integrating transcriptomic data and artificial intelligence to personalize curative treatments for head and neck cancer patients

**DOI:** 10.1038/s41698-026-01369-2

**Published:** 2026-03-14

**Authors:** Stefano Cavalieri, Loris De Cecco, Dario Monzani, Hisham Mehanna, Renata Ferrarotto, Christian Simon, Robert Haddad, Pierre Saintigny, Christophe Le Tourneau, Lisa Licitra

**Affiliations:** 1 Head and Neck Medical Oncology Department, Fondazione IRCCS Istituto Nazionale dei Tumori di Milano, Milan, Italy; 2 Department of Oncology and Hemato-oncology, University of Milan, Milan, Italy; 3 Integrated Biology of Rare Tumors, Department of Experimental Oncology, Fondazione IRCCS Istituto Nazionale dei Tumori, Milan, Italy; 4 Applied Research Division for Cognitive and Psychological Science, IEO, European Institute of Oncology IRCCS, Milan, Italy; 5 Department of Psychology, Educational Science and Human Movement, University of Palermo, Palermo, Italy; 6 Institute of Head and Neck Studies and Education (InHANSE), Dept of Cancer and Genomic Sciences, University of Birmingham, Birmingham, UK; 7 Department of Head and Neck Medical Oncology, The University of Texas MD Anderson Cancer Center, Houston, TX USA; 8 Department of Otolaryngology-Head and Neck Surgery, Centre Hospitalier Universitaire Vaudois (CHUV), UNIL, Lausanne, Switzerland; 9 Department of Medical Oncology, Division of Head and Neck Oncology, Dana-Farber Cancer Institute, Boston, MA USA; 10 Harvard Medical School, Boston, MA USA; 11 Department of Medicine, Brigham and Women’s Hospital, Boston, MA USA; 12 Department of Medical Oncology, Centre Léon Bérard, Lyon, France; 13 Univ Lyon, Claude Bernard Lyon 1 University, INSERM 1052, CNRS 5286, Centre Léon Bérard, Cancer Research Center of Lyon, Lyon, France; 14 Department of Drug Development and Innovation (D3i), Institut Curie, Paris, France; 15 INSERM U900 Research unit, Institut Curie, Paris, France; 16 Paris-Saclay University, Paris, France

**Keywords:** Cancer, Computational biology and bioinformatics, Oncology

## Abstract

Personalized treatment in head and neck cancer remains limited despite substantial biological heterogeneity. Using the SuPerTreat project as a case study, we outline a prototype clinical decision support system (CDSS) integrating transcriptomic data and artificial intelligence (AI), and summarize expert consensus on its potential, requirements for accuracy, validation, regulatory alignment, and clinical implementation. This Perspective provides a roadmap to guide future development and responsible integration of CDSS into precision oncology.

Precision medicine is rapidly advancing across various medical fields, fueled by the continuous evolution of Artificial Intelligence (AI) technologies. In oncology, precision medicine is revolutionizing patient outcomes by tailoring treatments to individual genomic profiles. Precision oncology embraces the integration of genetic alterations in the decision-making process. Treatment targeting a defined molecular alteration largely demonstrated clinical efficacy across various tumor types^1^. Consequently, comprehensive multigene sequencing has become a standard tool for diagnosis and treatment allocation^2^.

This field of innovation usually applies for patients with advanced recurrent/metastatic cancer. Unfortunately, in the treatment of loco-regionally advanced non-metastatic head and neck squamous cell carcinoma (HNSCC), where curative treatments include various combinations of surgery, radiotherapy, and chemotherapy^3,4^, the implementation of personalized oncology has been challenging and remains underdeveloped.

To improve outcomes in the HNSCC definitive setting, several clinical trials have explored both therapeutic intensification and de-intensification strategies^5,6^. Notably, these studies did not reach the goal of modifying the standard of care (SoC) for HNSCC patients. Escalating curative treatments with concomitant or adjuvant immunotherapy did not significantly improve survival in unselected patients^5–7^. Survival benefit for HNSCC patients undergoing surgery has been recently observed with neoadjuvant immune checkpoint inhibitors^8^.

Moreover, it is central to consider that HNSCC should not be considered and managed with a “one-size-fits-all” approach. The clinical heterogeneity is made more complex by the biological intricacy. Unlike other solid cancers, HNSCC typically lacks druggable genetic mutations or actionable molecular alterations^9^, making genomics a relatively poorly informative field for HNSCCs. Nevertheless, transcriptomics was found to be informative in this setting. Indeed, six gene expression (GE) clusters were described in HNSCC^10^, and such heterogeneity makes these malignancies potentially suitable for personalized treatments^11,12^.

The need for a customized approach is now even more pressing due to the failure of the current patient stratification by prognostic factors as clinical and biological features, consisting mainly of stage, site of disease, performance status, comorbidities, smoking history, and HPV status. One could speculate that failures in achieving improved outcomes and effective patient stratification can be attributed to several factors as prognosis determined from a relatively small number of cases and/or based on inadequate or insufficient criteria. Other drawbacks include relying solely on a good prognosis for de-intensification, whereas additional selection factors are necessary. Failures may be also due to the presence of subgroups with varying prognoses even within diseases with an overall favorable clinical behavior (e.g., three GE clusters in HPV+ oropharyngeal carcinoma)^13^.

This manuscript does not aim to present original validation results, but rather to propose a translational framework and expert-informed roadmap for the development, validation, and future clinical implementation of CDSS in curative HNSCC.

## AI and CDSS in oncology

Recent years have seen exponential growth in AI applications in oncology^14,15^. These new tools have the potential to improve the precision in diagnosis, prediction of outcomes, and to reach a tailored treatment^14,16^. Today, AI systems are increasingly being incorporated into several clinical settings, such as image-based computer-aided discovery and diagnosis (subtyping, grading, staging); translation of genomic information; prediction and tracking of patient’s response; and identification of pathological features^16,17^. By combining *omics* and phenotype data, AI can assist physicians in patient stratification, in clinical decision-making, and in the discovery of new biomarkers to predict the recurrence of disease^16,17^.

A significant number of publications using AI to identify novel diagnostic and prognostic biomarker signatures on tissue images of different cancer types are currently accessible^18^. In particular, these studies demonstrated that the identification of biomarkers and genetic modifications by AI is effective, especially in breast, gastrointestinal, prostate, and lung cancers^17^. In this scenario, most of the biomarkers showed a significant role in cancer diagnosis, tumor subtyping, evaluation of pathologic features, and only in some cases for prognosis forecasting^17^. Therefore, the interest in exploiting AI for biomarker development is crucial, but so far, limited interest has been assigned in the field of HNSCC amenable to curative treatments, which is the main use case scenario and focus of the current work.

Clinical decision support systems (CDSS) are tools that may integrate clinical data and features obtained through AI. The systems process data adequately, matching them to their database and reaching a recommendation that can be implemented in the decision-making process at the point of care and should be aligned with clinical practice guidelines^19^. An increasing number of applications of CDSSs in precision oncology have been described. Kumar and colleagues developed a machine learning CDSS capable of identifying predictive features in patients affected by breast cancer. The tool can select patients and predict cancer outcomes, outperforming the state-of-the-art strategy in precision, specificity, accuracy, and sensitivity^20^.

## CDSSs validation criteria

Despite their potential, only a few CDSSs have been externally validated and implemented in clinical practice^19^. CDSSs should satisfy numerous validation criteria to be safely implemented in daily care. Data are keys and should be interoperable, accurate, and available. Validity of recommendations and interoperability are the most important facilitators for implementation^19^. Hendriks and colleagues have built an algorithm to check the validity of CDSS aimed at ensuring the concordance between multidisciplinary teams’ decisions and CDSS recommendations^19^. The CDSS should also be made available for everyday clinical practice, aligned with the preferences of the clinicians involved and of the patients. Finally, it should be easy to use and transparent^19^. The main barriers to the implementation of CDSS are missing data, inability to reuse data, insufficient standardized definition, and time-consuming data collection^17^. Other hurdles include the failure of the CDSS to deliver real-time support and be integrated into the clinical workflow^19^.

## AI and CDSS in HNSCC

So far, the application of AI in the context of HNSCC has focused in the following areas: detection of precancerous and cancerous lesions in histopathologic slides; prediction of histopathologic nature of a lesion from imaging; prognostication; extraction of pathological findings from imaging; and applications in radiation oncology^21^.

Biological model applications in HNSCC have demonstrated excellent prognostic and potentially predictive powers. Indeed, an unsupervised analysis of the molecular heterogeneity of HNSCC revealed the existence of six GE subtypes. These molecular data provided useful information for the prognosis of HNSCC patients^10^.

Another prediction model has been developed in the field of HPV+ oropharyngeal squamous cell carcinoma^22^. The peculiarity of this model was the inclusion of genomic adjusted radiation dose (GARD), a previously described model of the treatment effect of radiotherapy, in outcomes prediction of patients treated with radiotherapy (+/− chemotherapy)^22^. In a virtual trial, GARD outclassed a clinical nomogram in predicting overall survival (OS). Moreover, GARD predicted the decrease of OS in association with RT dose de-escalation, and proposed a new method to stratify patients based on GE^22^.

## Translating the biological complexity into clinical practice—expert discussion

An international team of HNSCC specialists met on January 26th, 2024, in Milan, Italy, to discuss the challenges and opportunities of integrating biological complexity, particularly gene expression (GE), into clinical practice. They focused on a CDSS aimed to support personalized treatment decisions in HNSCC candidate to definitive treatments. This CDSS, developed within the SuPerTreat project (“Supporting Personalized Treatment Decisions in Head and Neck Cancer through Big Data”, funded by ERA PerMed, and the EU’s Horizon 2020 under the Marie Skłodowska-Curie Actions Grant), was used as a case study. The discussion highlighted the unmet needs for integrating CDSS-based decision-making into clinical practice, the potential for CDSS to modify therapeutic approaches, its acceptance among clinicians, and the required accuracy for reliable predictions.

## A novel CDSS in HNSCC management/case study—prototype description

In the present manuscript, SuPerTreat is not described in terms of model training, dataset composition, or clinical utility metrics, but rather as a prototype CDSS used to support a translational and expert-driven discussion. In this scenario, the SuPerTreat project led to the development of a CDSS to predict patient prognosis by integrating clinical and biological data. This collaborative European initiative aimed to validate methods that incorporate real-world data from transcriptomic analyses. The SuPerTreat CDSS leverages proprietary and public repositories of gene expression data to identify subpopulations that could benefit from specific treatments. The AI-based models developed under SuPerTreat allow the entry of different covariates for the survival analysis, such as dataset (proprietary vs public); genomic data, age, sex, TNM stage (I-II vs. III/IVa/IVb), smoking status (current/former vs. never), tumor subsite (oral cavity vs. HPV+ oropharynx vs. HPV- oropharynx vs. larynx vs. hypopharynx), treatment (surgery, radiotherapy, systemic treatment), setting (curative, rec/met). The endpoints considered are: overall survival (all patients); disease-free survival (curative setting only), and progression-free survival (rec/met setting only).

The models have produced validated prognostic and predictive GE signatures, which are incorporated into the CDSS as independent modules, without implying additive or synergistic performance at the current stage. To address skepticism about its usability,the prototype CDSS was qualitatively discussed by experts in terms of usability, interpretability, and potential clinical relevance; no formal quantitative consensus metrics were collected. The results showed that certain GE profiles could benefit more from specific therapies like radiation, surgery, or chemotherapy. The ultimate goal is to validate the SuPerTreat CDSS thoroughly to ensure its timely implementation in clinical practice in the future.

## Results of expert discussion/qualitative consensus

The following section summarizes qualitative insights derived from expert discussion rather than quantitative outcome analyses. To shape the discussion, the experts discussed the following clinical use case scenarios for HNSCC patients candidates to curative treatments:

1. HPV-negative HNSCC patient with a high-risk disease detected at tumor GE profiling. Would you accept to intensify the curative treatment? *E.g*., adding radiation for cases without indication as per current guidelines, or adding chemotherapy concomitantly with radiation for cases candidates to exclusive radiation as per current guidelines
2. Assuming an equivalence between surgery and radiotherapy for a specific HNSCC, would you accept the following schema based on tumor GE profiling? If biologic profiling reveals that the tumor is radiosensitive, the patient receives radiotherapy. If biologic profiling reveals that the tumor is radioresistant, the patient undergoes surgery.
3. HNSCC patient who is a candidate for radiation alone (either post-operative or definitive), meaning that no systemic therapies would be administered as per guidelines. GE profiling shows that the tumor has a profile characterized by suboptimal prognosis (compared to what may predicted by clinical characteristics) and a sensitivity to the drug cetuximab. Would you accept to intensify the curative treatment adding cetuximab to radiotherapy?

These three scenarios augmented the expert discussion, which elicited several thoughts and considerations that are reported herein. First of all, experts highlighted the urgent need for the application of AI in the integration of biological data in clinical decision-making in the context of definitive treatments for loco-regionally advanced HNSCC.

The discussion allowed to emphasize the potential of AI in oncology, not only in terms of outcome but also in training less experienced physicians in a better evaluation and decision process in HNSCC.

In clinical practice, a non-negligible fraction of HNSCC patients have poor outcomes, so it is crucial to implement transcriptomic data to guide clinical decisions in order to choose the best treatment. Some of the most important unmet needs are to know for which patient surgery or radiation, with or without chemotherapy, would be beneficial. All experts recognized the enormous potential value of SuPerTreat CDSS in HNSCC management.

Furthermore, several critical aspects emerged concerning the applicability of SuPerTreat CDSS in clinical practice that are discussed below.

### Regulation

All experts agreed that the use of CDSS in clinical practice needs a good clinical practice (GCP) / good clinical laboratory practice (GCLP) validation. Data in the CDSS comes from research and can be re-analyzed in clinical labs, and it is crucial to perform further validation. A European initiative will regulate and facilitate the sharing and the pulling of data across borders and institutions. This kind of initiative will cover data standardization, analysis models, and patients’ consent for the use of data.

### Accuracy

To discuss the accuracy, the experts considered the concordance index (Harrell’s C-index) in the prediction of 2-year overall survival of four GE models based on the clinical scenario of interest (Table 1). These C-index values are reported to illustrate the level of prognostic performance achieved by individual, previously validated GE models within their original clinical contexts, and are not intended to represent the performance of the integrated CDSS or to imply synergistic effects between models. Additional details regarding sample size, external validation, and clinical context of each model are summarized in Table 1.

**Table 1:** Clinical scenarios and previously published gene expression models used as use-cases

Clinical scenario	Gene expression model	Reference	C-index (2-year OS)	Sample size (original publication)	External validation	Clinical relevance
HPV-negative HNSCC patients	172GS	De Cecco L et al. 2014^23^	0.71	841 HNSCC	Yes	Prognostic stratification beyond TNM and HPV status
HNSCC patients treated with radiotherapy	RSI	Eschrich SA et al. 2009^24^ Torres-Roca JF et al. 2023^25^	0.70	>4000 (524 HNSCC)	Yes	Prediction of radiosensitivity and RT benefit
HNSCC patients treated with platinum-based chemotherapy	Pancancer-cisplatin	Wells JD et al. 2021^26^.	0.72	194 (65 HNSCC)	Yes	Identification of platinum-sensitive tumors
HNSCC patients treated with systemic treatment (with or without cetuximab)	Cl3-hypoxia	De Cecco L et al. 2015^10^	0.73	1386 HNSCC	Yes	Prognostic and treatment-related stratification

Reported performance metrics, sample sizes, and validation status refer to the original publications and do not represent the performance of the integrated CDSS.

Experts acknowledged that currently available gene expression models show prognostic performance around 70% in terms of concordance index (Table 1); however, there was broad agreement that substantially higher accuracy would be required to justify treatment intensification or de-intensification in routine clinical practice.

### Validation

Even if data have been already externally validated (i.e., only already published GE models were included in the CDSS), further validation steps are needed for the workflow, possibly through prospective or retrospective trials. The model will need to be tested as a new paradigm in clinical practice, comparing the use of the model compared to SoC. Results must be consistent with current knowledge. Beyond prognostic performance, future validation steps will need to formally quantify clinical net benefit, including decision curve analysis, comparative effectiveness, and health-economic impact, before the CDSS can be considered for routine clinical use.

### Clinical impact and health-economic considerations

Demonstrating clinical utility is a prerequisite for the implementation of CDSS in oncology. For SuPerTreat, this will require formal assessment of net clinical benefit, comparison with standard-of-care decision strategies, and evaluation of cost-effectiveness in different healthcare systems. These aspects are integral components of the proposed development roadmap but are not addressed in the present work.

### Simplicity and transparency

The experts found the tool simple to use. It is important that CDSS is understandable and will use performance indicators to monitor its performance, efficacy and accordance to current Guidelines.

Other aspects deemed worthy of attention are visualization, increasing patient numbers, trustworthiness, and data quality.

### Visualization

**Fig. 1:**
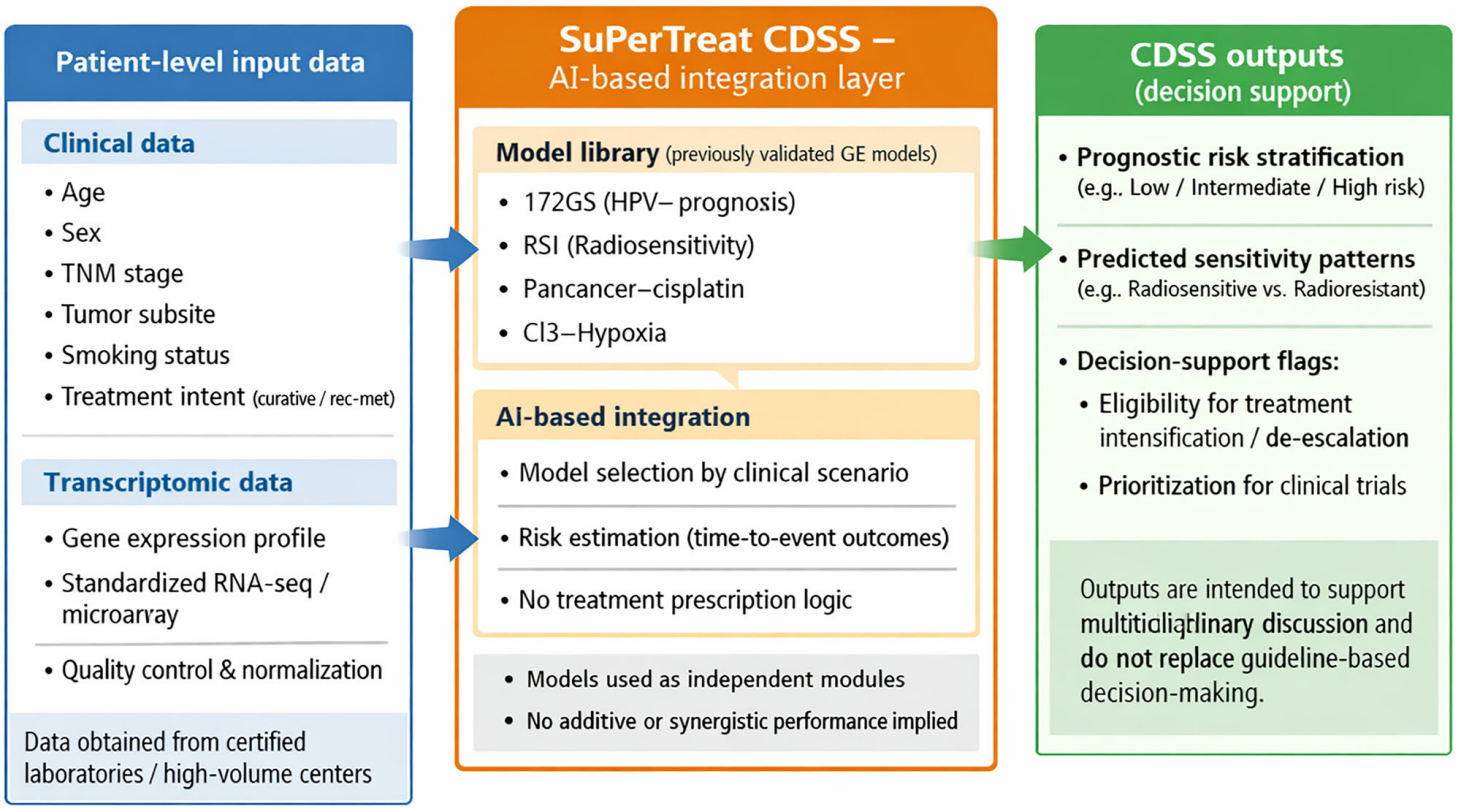
Schematic workflow of the SuPerTreat clinical decision support system (CDSS). Patient-level clinical variables and transcriptomic data are integrated within the SuPerTreat CDSS, which incorporates previously published and externally validated gene expression models as independent modules. The system generates prognostic stratification and decision-support outputs intended to complement multidisciplinary discussion and guideline-based care. The CDSS is presented as a prototype framework and requires formal clinical validation before routine clinical implementation.

A schematic overview of the end-to-end SuPerTreat CDSS workflow, from data input to decision-support output, is shown in Fig. 1. Three possible outputs of the CDSS were hypothesized: textual, bar graphs, and pictographs (Supplementary Fig. 1, panels A, B, and C, respectively). Experts agreed on the fact that the recommendation must be simple and immediately clear both for clinicians and patients. Therefore, a bar graph was deemed as an ideal example.

### Acceptability

There was broad consensus that we will need a strategy to manage challenges in clinician acceptance, for example emphasizing the potential benefits in healthcare costs.

### Increasing patient numbers

Experts consistently emphasized that accuracy depends mostly on the number of patients and statistics. Strategies will be initiated to involve pharmaceutical companies and international groups to expand patient data.

### Data quality

All experts agreed that data should come from high-volume and high-reputation centers, where they perform both genomic expression tests and therapies.

### Trustworthiness

The method should be transparent. Recommendations have to be perceived as replicable and consistent with current knowledge.

### Summary of consensus statements

- CDSS integrating transcriptomic data has strong potential to improve patient stratification in curative HNSCC.
- Such systems should not be used to modify standard treatments outside clinical trials until formal validation is completed.
- Accuracy thresholds substantially higher than current clinical models are required to justify treatment intensification or de-intensification.
- Prospective validation and health-economic assessment are mandatory before clinical implementation.

## Limitations

This work does not include independent retrospective or prospective validation of the SuPerTreat CDSS, nor formal comparative analyses against standard prognostic models. Therefore, the system should be considered a prototype and hypothesis-generating tool. Rigorous clinical validation, health-economic evaluation, and regulatory assessment are required before clinical adoption.

## Proposed implementation roadmap for CDSS in curative HNSCC

*Short-term (current stage)*

- Integration of previously validated transcriptomic models
- Standardization of data pipelines and outputs
- Retrospective benchmarking against standard-of-care decision pathways

*Mid-term (1–3 years)*

- Prospective observational validation
- Decision-analytic and health-economic evaluation
- Regulatory alignment and good clinical practice and good clinical laboratory practice (GCP/GCLP) compliance

*Long-term (3–5 years)*

- Interventional clinical trials guided by CDSS output
- Reimbursement strategies and payer engagement
- Full integration into multidisciplinary tumor boards

## Conclusion

The integration of biological and clinical data enhances prognostic accuracy in HNSCC patients. GE is particularly promising, providing more precise prognostic information than clinical or pathological data, including HPV status. The SuPerTreat CDSS illustrates the potential role of transcriptomic-driven decision support systems in improving patient stratification strategies in the curative setting. Incorporating GE models into clinical research and practice will influence clinical trial ethics, cost-effectiveness, and serve educational purposes for patient information and empowerment.

Currently, modifying standard therapies based solely on biological prognostic models, even those that are highly accurate and externally validated, is not SoC. However, benchmark studies have shown benefits from treatment adjustments guided by these models. Furthermore, such models can help select clinical trial participants more effectively, potentially reducing the number of patients required for studies. Integrating GE and clinical data through tools like the SuPerTreat CDSS is a promising strategy for personalizing curative treatments in HNSCC. Stringent validation studies are needed to support its broader implementation in research and clinical settings. This work should be interpreted as a roadmap and consensus framework rather than as evidence of clinical utility. Demonstrating additive or synergistic prognostic value through formal comparative metrics will be a critical component of future validation studies. Engaging academic institutions in data sharing will be crucial for advancing clinical practice in HNSCC management.

## Supplementary information

Supplementary Information

## Data Availability

No datasets were generated or analysed during the current study.
