# Supplementary material for: Integrating transcriptomic data and artificial intelligence to personalize curative treatments for head and neck cancer patients
Source: NPJ Precis Oncol. 2026 Mar 14;10:170. doi: 10.1038/s41698-026-01369-2 (PMC13133119; doi:10.1038/s41698-026-01369-2)
Supplement: Supplementary file 1 — Supplementary Information [file 41698_2026_1369_MOESM1_ESM.pdf]

A

Your 2-year survival rate is 98%. This means that out of every 100 people treated for head and neck cancer with radiotherapy and concomitant chemotherapy, 98 will survive for at least 2 years after treatments

B

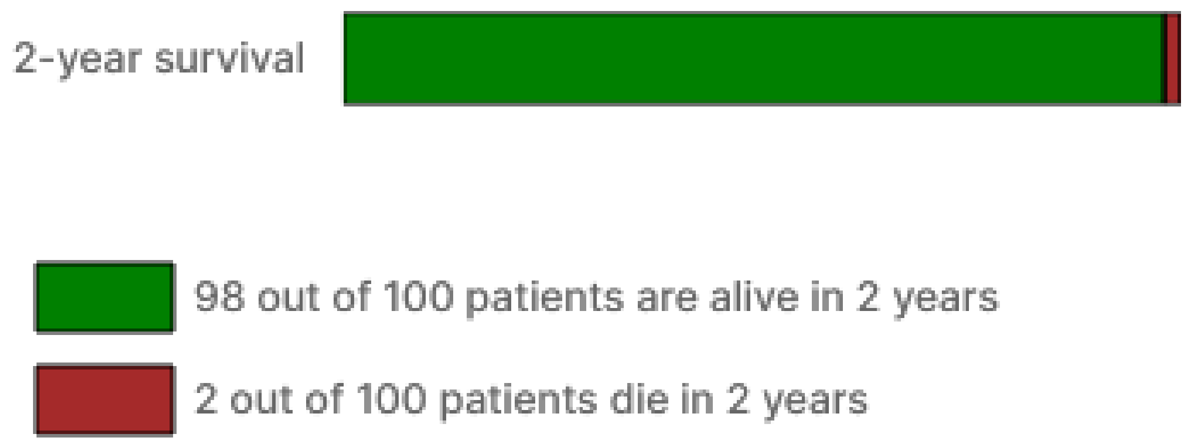

C

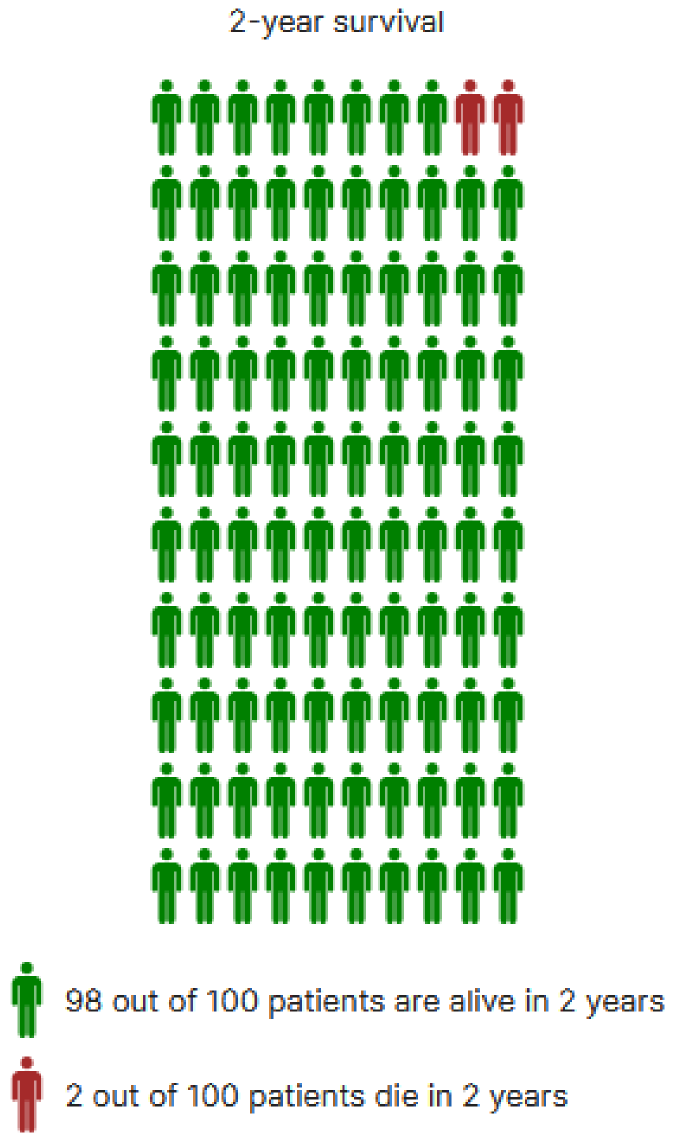

Supplementary Figure 1. CDSS outputs of the CDSS: textual (A), bar graphs (B), and pictographs (C).
